# SEER-based risk stratification system for patients with primary non-cirrhotic liver cancer

**DOI:** 10.1007/s00432-023-05057-7

**Published:** 2023-07-08

**Authors:** Runmin Cao, Honghe Jiang, Guangpeng Liang, Weibin Zhang

**Affiliations:** 1 Graduate School, Jinzhou Medical University Postgraduate Training Base (Jinzhou Central Hospital), Jinzhou City, Liaoning Province China; 2 Department of Clinical Medicine, Anhui University of Science and Technology, Huainan City, Anhui Province China; 3 Department of General Surgery, Jinzhou Medical University Postgraduate Training Base (Jinzhou Central Hospital), Jinzhou City, Liaoning Province China

**Keywords:** Hepatocellular carcinoma, Non-cirrhotic liver, Nomogram, Overall survival, Risk stratification system

## Abstract

**Background:**

Little research has been done on the factors affecting the survival of patients with non-cirrhotic hepatocellular carcinoma (HCC-NCL). Our aim was to develop and validate a nomogram and a new risk stratification system that can evaluate overall survival (OS) in HCC-NCL patients.

**Methods:**

We retrospectively analyzed data from the Surveillance, Epidemiology, and End Results (SEER) database from 2010 to 2019 to study HCC-NCL patients. The patients were randomly split into training and validation groups at a 7:3 ratio and subjected to single-factor and multi-factor COX regression analysis. We then developed a nomogram and evaluated its accuracy and clinical validity using time-dependent ROC, DCA, and calibration curves. We compared the nomogram with the AJCC staging system by calculating C-index, NRI, and IDI. Finally, we used Kaplan–Meier curves to compare the nomogram and AJCC staging. These analyses were performed without altering the original intended meaning.

**Results:**

AFP levels, surgical intervention, T-stage, tumor size, and M-stage were independent prognostic indicators for overall survival among the HCC-NCL population studied. We developed a nomogram based on these factors, and time-dependent ROC, calibration curves, DCA analyses, and C-index proved its accuracy. Compared to the AJCC staging system, the nomogram showed better prognostic accuracy through time-dependent ROC, DCA analyses, C-index, NRI, IDI, and Kaplan–Meier curves.

**Conclusion:**

We have developed and validated a survival nomogram applicable to HCC-NCL patients, with risk stratification. Our nomogram offers personalized treatment and management options superior to those provided by the AJCC staging system.

**Supplementary Information:**

The online version contains supplementary material available at 10.1007/s00432-023-05057-7.

## Introduction

With a rising incidence, hepatocellular carcinoma now ranks as the fifth most commonly occurring tumor around the world (Wen et al. 2022). Alarmingly, it is additionally the second most common reason for cancer-related deaths (Rumgay et al. 2022). Cirrhosis is the primary causal factor in hepatocellular carcinoma patients, accounting for over 80% of cases (Q-song et al. 2021). Consequently, both the National Comprehensive Cancer Network (NCCN) and the European Association for the Study of the Liver (EASL) guidelines utilize cirrhosis as a factor for risk stratification in relation to the development of hepatocellular carcinoma (Galle et al. 2018; Benson et al. 2021). An increasing number of studies have revealed variations in clinical examination and treatment approaches between patients with hepatocellular carcinoma without cirrhosis and those with cirrhotic hepatocellular carcinoma, potentially resulting in varying prognostic risk factors and survival lengths. Initially, cirrhosis causes liver tissue to lose its normal structure and physiological functions (Baglieri et al. 2019), resulting in non-cirrhotic hepatocellular carcinoma patients who often exhibit no clinical symptoms during the early stages and are usually diagnosed at an advanced stage due to their well-preserved liver function and a lack of surveillance imaging (Desai et al. 2019). Second, patients diagnosed with non-cirrhotic hepatocellular carcinoma (HCC-NCL) experience a lower incidence of perioperative complications and mortality when compared to those with cirrhotic hepatocellular carcinoma (Zhou et al. 2014). Furthermore, patients with cirrhotic hepatocellular carcinoma are more responsive to systemic therapy with medication (Desai et al. 2019). Lastly, individuals with HCC-NCL are capable of undergoing repeat second and third liver resections in case of tumor relapse, which is as safe and effective as their initial surgery (Gaddikeri et al. 2014). Regrettably, the majority of present research has predominantly concentrated on the prognostic analysis of cirrhotic hepatocellular carcinoma, with little exploration into the factors that impact survival in HCC-NCL. While AJCC staging is widely utilized for assessing tumor prognosis, including hepatocellular carcinoma (Sirivatanauksorn and Tovikkai 2011), it still maintains limitations as a system that solely incorporates the anatomical aspects of the tumor (Maida 2014). This renders it more adept at evaluating tumor spread and less capable of assessing prognosis. A novel model is essential for accurately assessing the prognosis of patients with HCC-NCL. In current study, utilizing a more extensive sample size obtained from the SEER database, we developed a nomogram and risk stratification system to predict patient survival in those with HCC-NCL. By doing so, clinicians can expedite clinical decision-making and prompt early clinical intervention for patients diagnosed with cirrhosis-free hepatocellular carcinoma, thereby extending their lifespan.

## Materials and methods

### Data gathering

We collected clinical information on hepatocellular carcinoma patients from 2010 to 2019 using SEER*Stat 8.4.0.1, encompassing information from 17 different centers. Institutional ethics committee approval was deemed unnecessary as the SEER database has removed all private information from the patient data collected.

### Data collation

Inclusion criteria: (1) hepatocellular carcinoma (HCC) selected using ICD-O-3 histological codes (8170-3, 8171-3, 8172-3, 8173-3, 8174-3, 8175-3); (2) HCC patients without cirrhosis (Ishak 0-4; No to moderate fibrosis; METAVIR F0-F3; Batt-Ludwig 0-3). Exclusion criteria: (1) race information unknown, (2) marital information unknown, (3) age less than 19 years or more than 100 years, (4) non-first primary tumor, (5) not pathologically confirmed by microscopy, (6) histological grade unknown, (7) SEER grade unknown, (8) T-, N-, M-stage unknown, (9) surgical information unknown, (10) tumor size unknown, (11) survival information unknown. A total of 15 covariates were selected for analysis in this study, namely age, marital status, gender, race, AFP, radiotherapy, chemotherapy, systemic therapy, surgery, T-stage, N-stage, M-stage, tumor size, AJCC stage, and histologic grade. All patients were staged according to AJCC 8th edition. The patients were categorized into three groups based on the RX Summ–Surg Prim Site (1998+) field. These groups include: (1) absence of surgery (A000); (2) local destruction of tumor (A100–A170); and (3) liver resection or liver transplantation (A200–A750).

### Nomogram plotting and validation

After screening, we divided patients into training and validation groups, maintaining a 7:3 ratio. We screened independent risk factors using a stepwise backward COX multi-factor regression in the training group and subsequently constructed a nomogram. To evaluate the nomogram's precision, we utilized the c-index and area under the receiver operating characteristic (ROC) curve to assess its ability to predict outcomes. In addition, we employed the calibration curve to evaluate the precision of the nomogram and decision curve analysis (DCA) to evaluate the nomogram's net clinical benefit.

### New risk stratification system

The X-tile software is widely adopted for selecting cutoff values in several studies. In this study, the X-tile software was utilized to designate high, medium, and low risk to the total scores derived from the nomogram. Using the Kaplan–Meier approach, survival curves were plotted to compare different AJCC staging and diverse nomogram risk stratification.

### Data analysis

Categorical variables in the training and validation groups were presented as percentages and compared using the Chi-square test. Univariate regression of COX was screened for factors with *P* values < 0.05 and included in stepwise backward multivariate analysis. Independent risk factors were identified using Akaike information criterion (AIC), and nomogram was created. To test for multicollinearity, independent risk factors were assessed using variance inflation factors (VIF). The nomogram’s predictive ability, precision, and clinical usefulness were evaluated by utilizing the area under the ROC curve (AUC), c-index, calibration curve, and DCA curve. The predictive ability of the nomogram was compared to AJCC using the NRI, IRI, and C-index. We grouped the total nomogram score into high-risk, intermediate-risk, and low-risk categories, and compared the survival stratification ability of the nomogram to that of AJCC staging using the Kaplan–Meier method. We analyzed all statistical data using R software version 4.2.2, and performed two-tailed statistical tests to obtain *P* values. We considered *P* values less than 0.05 to be statistically significant.

## Results

### Patient characteristics

**Fig. 1:**
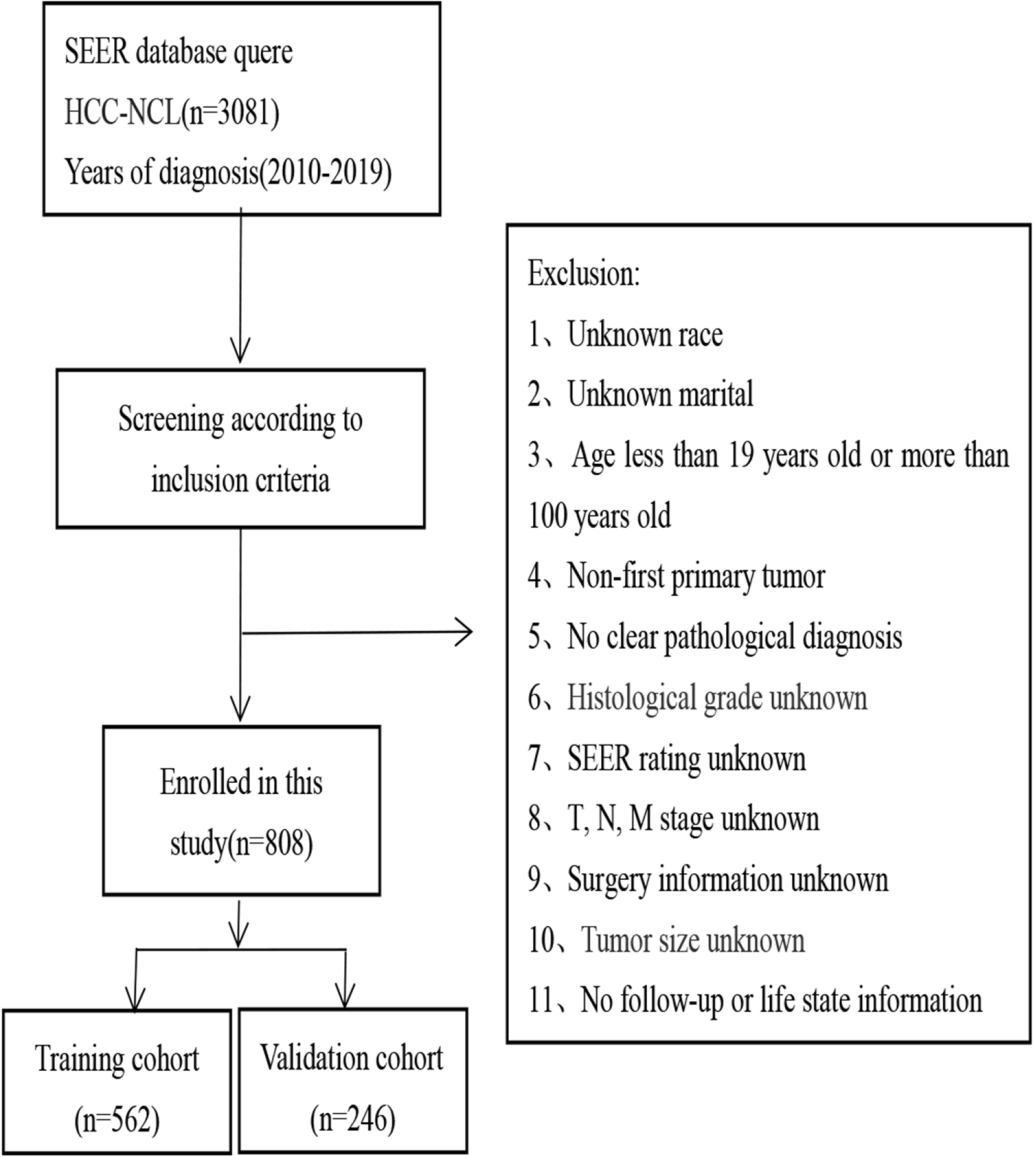
The study’s flow chart

Our study analyzed 808 non-cirrhotic hepatocellular carcinoma patients, including 562 (70%) in the training group and 246 (30%) in the validation group. Figure 1 depicts the study’s flow chart, while Table 1 presents the baseline characteristics of patients in both cohorts. Our analysis demonstrated no notable statistical distinctions between the training and validation cohorts.

**Table 1:** Baseline patient characteristics for both the training and validation cohorts

Characteristic	Whole population (*n* = 808)		Training cohort (*n* = 562)		Validation cohort (*n* = 246)		*P*
	Count	%	Count	%	Count	%	
Age							0.268
≤ 65 years	451	55.8	306	54.4	145	58.9	
> 65 years	357	44.2	256	45.6	101	41.1	
Race							0.790
White	469	58.0	328	58.4	141	57.3	
Black	83	10.3	55	9.8	28	11.4	
Other	256	31.7	179	31.9	77	31.3	
Sex							0.981
Male	613	75.9	427	76.0	186	75.6	
Female	195	24.1	135	24.0	60	24.4	
Marital							0.282
Single	285	35.3	191	34.0	94	38.2	
Married	523	64.7	371	66.0	152	61.8	
AFP							0.838
Negative	277	34.3	190	33.8	87	35.4	
Positive	425	52.6	296	52.7	129	52.4	
Unknown	106	13.1	76	13.5	30	12.2	
AJCC stage							0.986
I	414	51.2	286	50.9	128	52.0	
II	161	19.9	112	19.9	49	19.9	
III	157	19.4	110	19.6	47	19.1	
IV	76	9.5	54	9.6	22	8.9	
T-stage							0.909
T1	428	53.0	293	52.1	135	54.9	
T2	169	20.9	119	21.2	50	20.3	
T3	122	15.1	87	15.5	35	14.2	
T4	89	11.0	63	11.2	26	10.6	
N-stage							0.624
N0	769	95.2	533	94.8	236	95.9	
N1	39	4.8	29	5.2	10	4.1	
M-stage							1.000
M0	751	92.9	522	92.9	229	93.1	
M1	57	7.1	40	7.1	17	6.9	
Grade							0.571
I–II	612	75.7	422	75.1	190	77.2	
III–IV	196	24.3	140	24.9	56	22.8	
Surgery							0.536
No	224	27.7	151	26.9	73	29.7	
Local treatment	70	8.7	52	9.3	18	7.3	
Hepatectomy/transplant	514	63.6	359	63.9	155	63.0	
Radiation							0.096
No	732	90.6	516	91.8	216	87.8	
Yes	76	9.4	46	8.2	30	12.2	
Chemotherapy							0.484
No	586	72.5	403	71.7	183	74.4	
Yes	222	27.5	159	28.3	63	25.6	
Systemic							0.720
No	706	87.4	489	87.0	217	88.2	
Yes	102	12.6	73	13.0	29	11.8	
Tumor size							0.186
≤ 5 cm	374	46.3	251	44.7	123	50.0	
> 5 cm	434	53.7	311	55.3	123	50.0	

### Identification of independent risk factors

According to the results of univariate COX regression analysis, age, alpha fetal protein (AFP), pathological grade, radiotherapy, chemotherapy, surgical treatment, T-stage, tumor size, N-stage, and M-stage were found to have a significant impact on prognosis. Multifactorial analysis showed that the model with pathological grading, AFP, surgical treatment, T-stage, tumor size, and M-stage had the smallest AIC values, but since pathological grading significantly affects overall survival, we included AFP, surgical treatment, T-stage, M-stage, and tumor size as influencing survival of non-cirrhotic hepatocellular carcinoma (HCC-NCL) patients independent prognostic factors and entered into a columnar graphical model for analysis (Table 2). The results from the variance inflation factor (VIF) analysis indicated no evidence of multicollinearity among the variables included in multiple regression Model 2 (Additional file: Table S1: variance inflation factor of variable).

**Table 2:** COX regression analysis of overall survival in HCC-NCL patients

Characteristic	Univariate regression model		Multivariate regression Model 1		Multivariate regression Model 2	
	HR (95% CI)	P	HR (95% CI)	*P*	HR (95% CI)	*P*
Age			NI		NI	
< = 65	ref.					
> 65	2.019 (1.351–3.016)	< 0.001				
Race			NI		NI	
White	ref.					
Black	1.428 (0.988–2.065)	0.058				
Other	0.851 (0.659–1.098)	0.214				
Gender			NI		NI	
Male	ref.					
Female	0.821 (0.622–1.082)	0.161				
Marital			NI		NI	
Single	ref.					
Married	0.842 (0.665–1.067)	0.155				
Grade					NI	
I–II	ref.		ref.			
III–IV	1.476 (1.148–1.899)	0.002	1.243 (0.947–1.633)	0.117		
AFP						
Negative/normal	ref.		ref.		ref.	
Positive/elevated	1.655 (1.275–2.147)	< 0.001	1.552 (1.176–2.048)	0.002	1.615 (1.230–2.120)	< 0.001
Not document/unknown	1.141 (0.774–1.682)	0.504	1.440 (0.970–2.139)	0.070	1.467 (0.989–2.176)	0.057
Radiation			NI		NI	
No	ref.					
Yes	2.019 (1.351–3.016)	0.001				
Chemotherapy			NI		NI	
No	ref.					
Yes	1.947 (1.536–2.469)	< 0.001				
Systemic			NI		NI	
No	ref.					
Yes	1.302 (0.951–1.785)	0.100				
Surgery						
No	ref.		ref.		ref.	
Local tumor destruction	0.370 (0.24–0.568)	< 0.001	0.795 (0.490–1.290)	0.353	0.807 (0.498–1.310)	0.386
Hepatectomy/transplant	0.283 (0.221–0.363)	< 0.001	0.401 (0.303–0.531)	< 0.001	0.416 (0.316–0.548)	< 0.001
T-stage						
T1	ref.		ref.		ref.	
T2	1.631 (1.209–2.200)	0.001	1.597 (1.181–2.159)	0.002	1.596 (1.180–2.158)	0.002
T3	3.121 (2.293–4.248)	< 0.001	1.781 (1.245–2.548)	0.002	1.737 (1.217–2.483)	0.002
T4	5.250 (3.741–7.367)	< 0.001	2.394 (1.612–3.556)	< 0.001	2.510 (1.697–3.716)	< 0.001
N-stage			NI		NI	
N0	ref.					
N1	3.547 (2.282–5.515)	< 0.001				
M-stage						
M0	ref.		ref.		ref.	
M1	4.940 (3.415–7.146)	< 0.001	2.403 (1.619–3.567)	< 0.001	2.489 (1.680–3.688)	< 0.001
Tumor size						
< = 5 cm	ref.		ref.		ref.	
> 5 cm	2.233 (1.756–2.840)	< 0.001	1.635 (1.218–2.193)	0.001	1.673 (1.249–2.240)	< 0.001
AIC			3265.2		3265.6	

### Construction and validation of nomogram

**Fig. 2:**
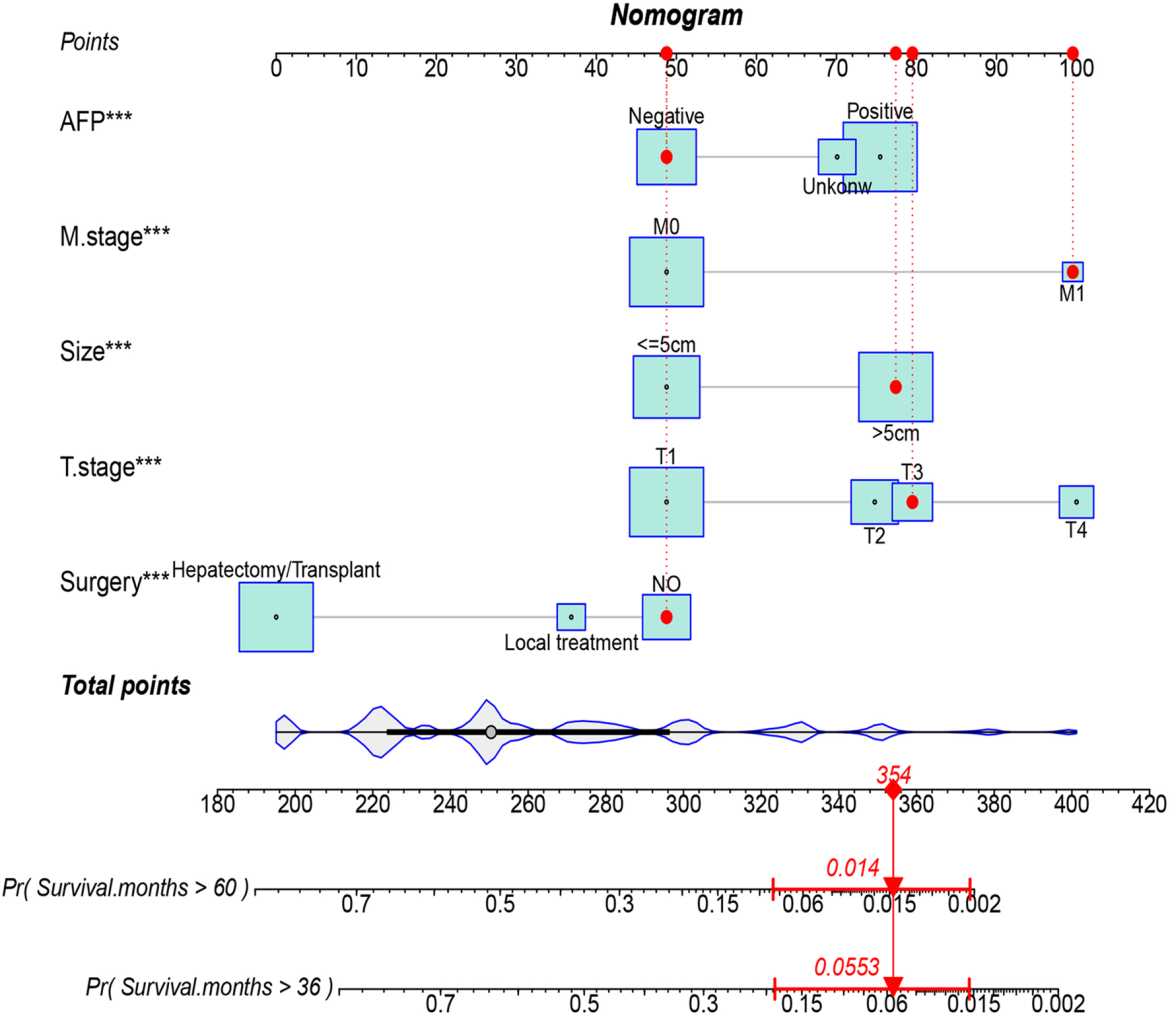
A nomogram for non-cirrhotic hepatocellular carcinoma patients

**Fig. 3:**
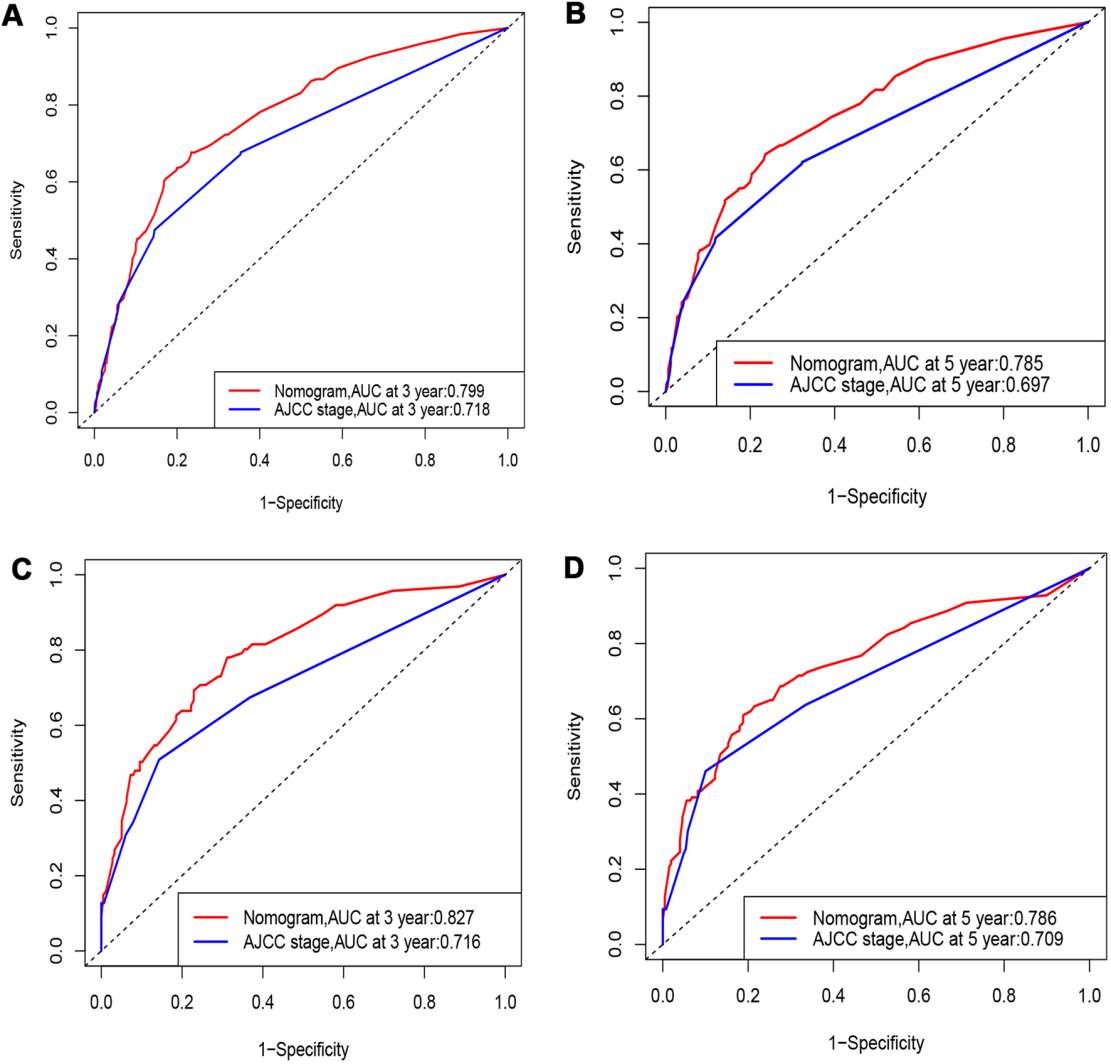
ROC of the nomogram. ** A** ROC curves for the training group at 3 years.** B** ROC curves for the training group at 5 years.** C** ROC curves for the validation group at 3 years.** D** ROC curves for the validation group at 5 years

**Fig. 4:**
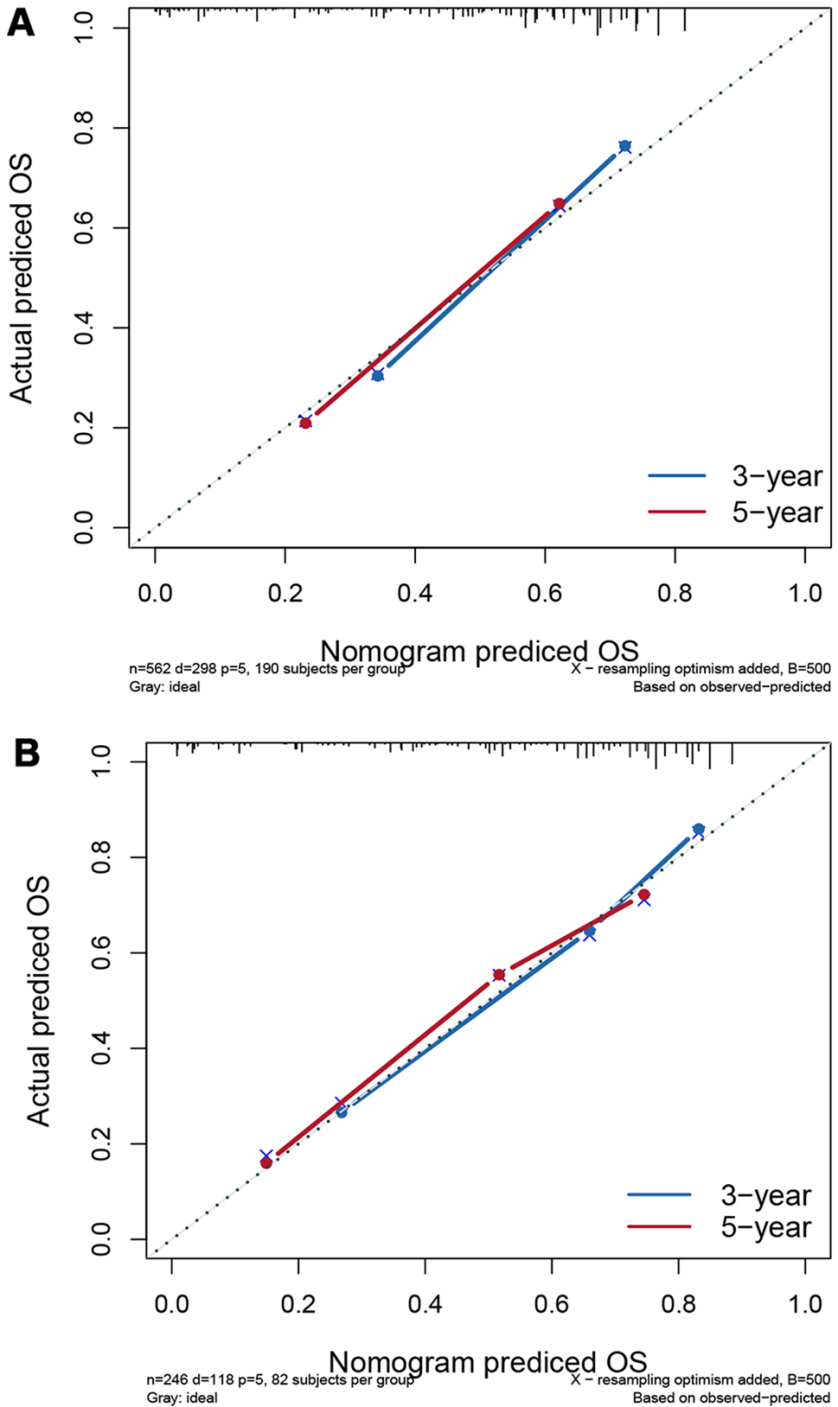
Calibration curves of training group and validation group. **A**Calibration curve of training group. **B** Calibration curve of validation group

**Fig. 5:**
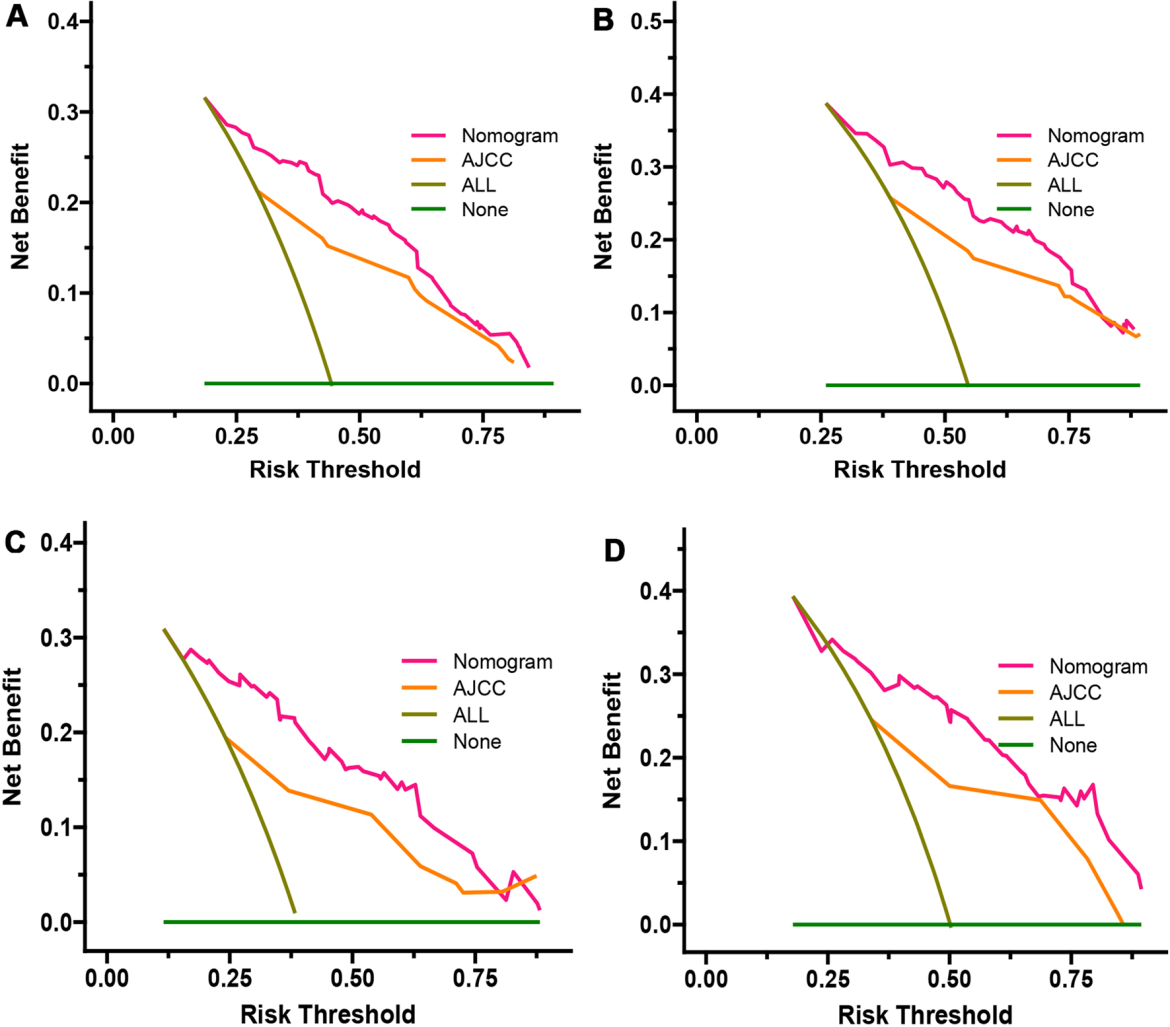
DCA curve of training group and validation group. **A** DCA curve of training group for 3 years. **B** DCA curve of training group for 5 years. **C** DCA curve of validation group for 3 years. **D** DCA curve of validation group for 5 years

In this study, we identified five variables (AFP, surgical treatment, T-stage, tumor size, and M-stage) that were used to construct nomogram for predicting the prognosis of HCC-NCL (Fig. 2). First, the risk score for each variable was calculated based on patient information, and then the scores of all variables were added together to obtain a total risk score for the patient (Additional file: Table S2: the score of each variable). Lastly, a straight line can be drawn in the final row, with the probability of 3-year and 5-year overall survival for patients with HCC-NCL serving as a reference for drawing the line. The nomogram we screened demonstrated excellent predictive performance, with C-indexes of 0.746 (95% confidence interval: 0.719–0.774) and 0.760 (95% confidence interval: 0.714–0.806) for the training and validation cohorts. Figs. 3, 4 and 5 demonstrate the ROC curves, calibration curves, and DCA curves. The AUC values for overall survival predictions of 3-year and 5-year in the training cohort were 0.799 and 0.785. In the validation cohort, the AUC values for 3-year and 5-year overall survival were 0.827 and 0.786. These results indicate the outstanding performance of the model in predicting overall survival in non-cirrhotic hepatocellular carcinoma patients. Furthermore, the DCA curves for both the training and validation cohorts associated with the screened 3- and 5-year nomogram demonstrated the positive net benefit and potential for clinical application. The calibration curves for both cohorts demonstrated a strong concordance between the predicted and actual probabilities of 3-year and 5-year overall survival.

### Comparison of the application value of nomogram and AJCC

**Fig. 6:**
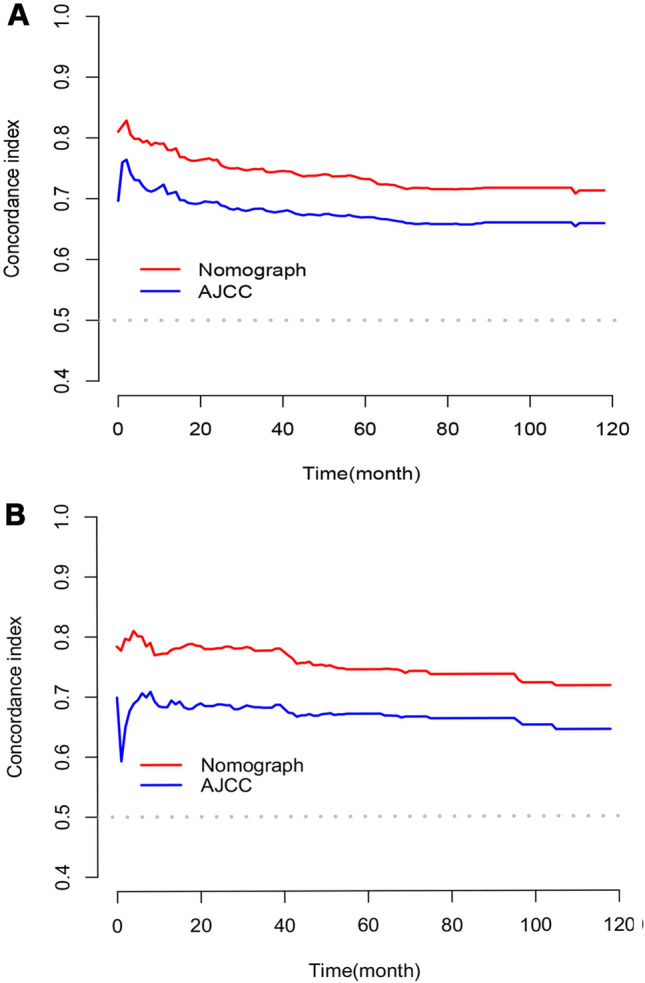
C index of nomogram and AJCC staging. **A** C index comparison between nomogram and AJCC staging in training group. **B** C index comparison between nomogram and AJCC staging in validation group.

The nomogram demonstrated superior C-index values compared to the AJCC staging model in both the training and validation groups, as depicted in Fig. 6. In the training cohort, the nomogram outperformed the AJCC staging model by exhibiting higher continuous net reclassification improvement (NRI) values of 0.273 and 0.174 for predicting 3-year and 5-year overall survival, respectively. The IDIs for 3-year and 5-year overall survival were also higher in the nomogram, with values of 0.072 and 0.066, respectively, in contrast to the AJCC staging model. Similarly, the validation cohort illustrated higher continuous NRI values for predicting 3-year and 5-year overall survival in the nomogram, with values of 0.333 and 0.251, respectively, compared to the AJCC staging model. Moreover, the IDIs for both 3-year and 5-year overall survival were also higher in the nomogram, with values of 0.125 and 0.105, respectively. These results demonstrate that the developed nomogram significantly outperformed the AJCC staging system, as indicated in Table 3. Additionally, the nomogram demonstrated higher AUC values compared to the AJCC staging model in both the training and validation cohorts, as shown in Fig. 3. This suggests that the nomogram model had superior accuracy over the AJCC staging criteria. The decision curve analysis (DCA) curves for both the training and validation cohorts provided robust evidence that the nomogram outperformed the AJCC staging model in predicting 3-year and 5-year overall survival, as indicated by the higher net benefits (Fig. 5).

**Table 3:** NRI and IDI compared to nomogram and AJCC staging criteria

Index	Training cohort			Validation cohort		
	Estimate	95% CI	*P* value	Estimate	95% CI	*P* value
Continuous NRI (vs. AJCC staging)						
For 3‐year OS	0.273	0.146–0.359	< 0.001	0.333	0.189–0.500	0.004
For 5‐year OS	0.174	0.106–0.370	0.004	0.251	0.109–0.440	0.004
IDI (vs. AJCC staging)						
For 3‐year OS	0.072	0.039–0.120	< 0.001	0.125	0.063–0.213	< 0.001
For 5‐year OS	0.066	0.033–0.109	< 0.001	0.105	0.047–0.195	< 0.001

### New risk stratification

**Fig. 7:**
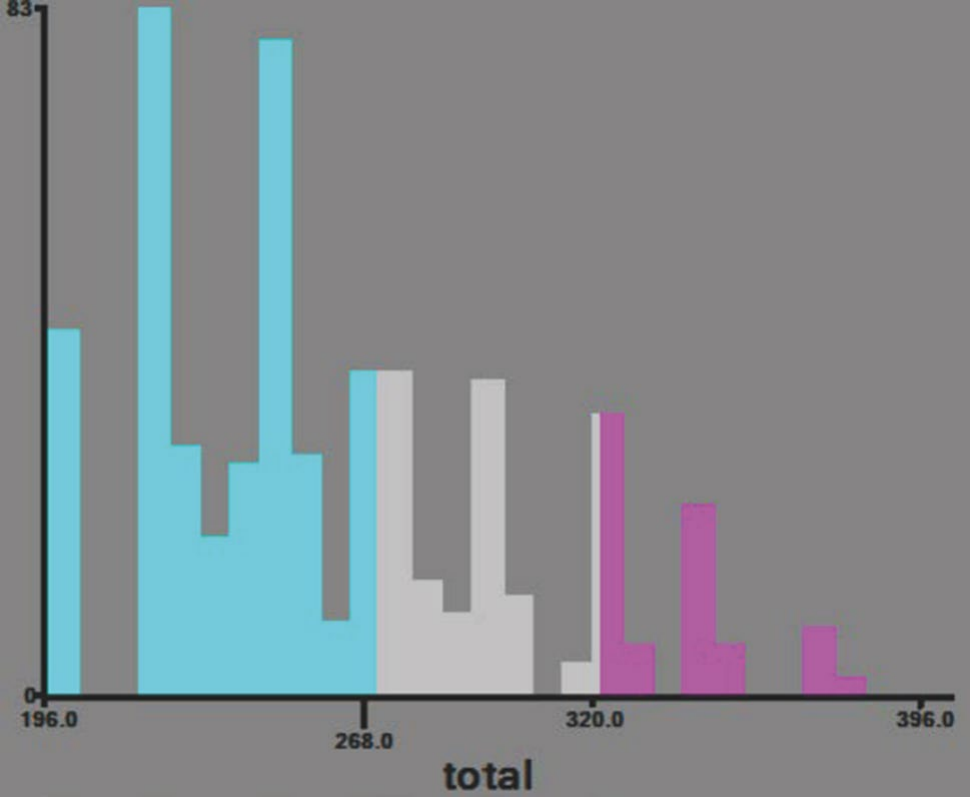
Cutoff point chosen for risk ranking via X-tile

**Fig. 8:**
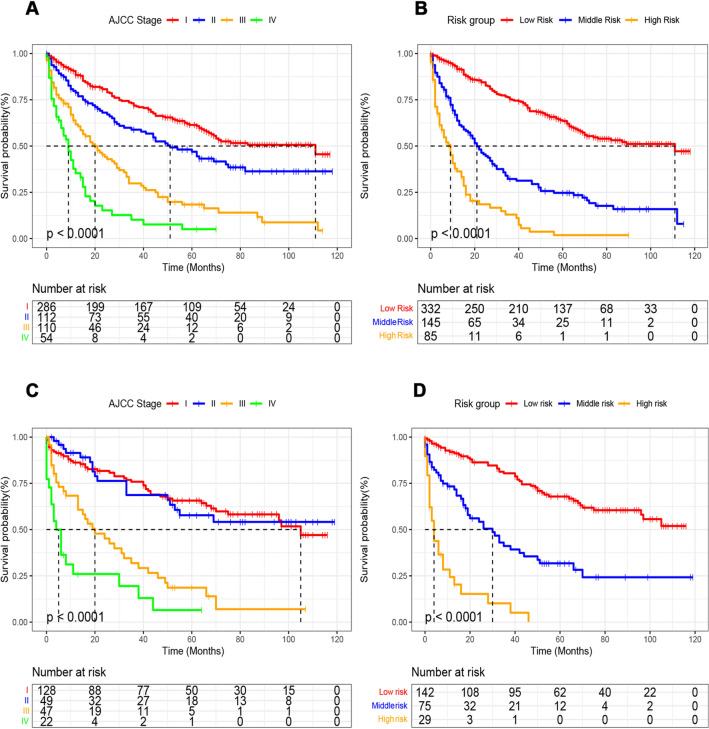
Kaplan-Meier OS curves for different risk stratification. **A, B** Kaplan–Meier OS curves of training and validation cohorts based on the new risk stratification system, **C, D** Kaplan–Meier OS curves of training and validation cohorts based on AJCC staging criteria

Through the application of the nomogram, we developed a novel risk stratification system that classifies patients based on their total score. The established nomogram classified patients into low-risk (total score ≤ 262), middle-risk (262 < total score ≤ 307), and high-risk (total score > 307) categories, as depicted in Fig. 7. The corresponding Kaplan–Meier curves displayed significant variations in overall survival rates across the three risk groups. In contrast, AJCC staging showed a limited capacity to distinguish between patients of different stages, indicating its inferiority in terms of prognostic stratification compared to nomogram. (Fig. 8).

## Discussion

The prevalence of hepatocellular carcinoma, the most frequent form of primary liver cancer, has been increasing in recent years (Rajendran et al. 2022). According to risk factors, hepatocellular carcinoma can be classified into two types: non-cirrhotic hepatocellular carcinoma (HCC-NCL) and cirrhotic hepatocellular carcinoma. The latter is more common, representing 80% of all hepatocellular carcinoma patients, while the former accounts for only 20% (Lee and Lee 2017). In comparison to cirrhotic hepatocellular carcinoma, patients with HCC-NCL are more likely to undergo resection and have a better overall survival rate despite having larger tumors (Yen et al. 2021; Gawrieh et al. 2019; Karaogullarindan et al. 2023). Moreover, the prognostic factors that affect hepatocellular carcinoma with or without cirrhosis are different (Karaogullarindan et al. 2023). Determining prognostic risk factors is crucial for managing patients and choosing the appropriate treatment approach. Identifying risk factors that affect survival time in HCC-NCL can be of great value. In this population-based study, 808 patients diagnosed with primary HCC without cirrhosis were evaluated, and it was shown that AFP positivity/elevation, liver resection/transplantation, T-stage, tumor size, and M-stage were independent risk factors for HCC-NCL. A novel nomogram and risk stratification system were developed based on the five significant risk factors. The system exhibited an excellent performance in predicting overall survival of HCC-NCL patients and also demonstrated its proficiency in accurately stratifying patients into different risk groups.

Previous studies examining multiple predictors of survival in HCC-NCL have reported controversial results. Nonetheless, this study identified larger tumors as a risk factor for survival in HCC-NCL, consistent with several other studies (Q-song et al. 2021; Yip et al. 2013; Wörns et al. 2012; Dasari et al. 2020; Penzkofer et al. 2022; Demirtas et al. 2021; Arnaoutakis et al. 2013). As tumor prognosis gains more attention, more and more tumors at substantial sites are incorporating mass size into AJCC staging. A larger tumor size correlates with increased energy consumption and more severe cancer-related weight loss, which can exacerbate cachexia in patients. Additionally, larger tumors can compress important blood vessels, nerves, or organs, further compromising the patient’s already weakened physiological functions. Larger tumors also decrease the likelihood that a patient will undergo radical surgery. Nevertheless, previous retrospective studies have not consistently identified larger tumors as a risk factor impacting survival in patients with HCC-NCL (Demirtas et al. 2021; Verhoef et al. 2004; Schütte et al. 2014; Xu et al. 2008). These discrepancies may be related to variances in patient characteristics and differences in sample sizes. Consistent with previous research, our study found that distant metastases, as well as advanced T-stage and M-stage, were identified as independent risk factors contributing to higher mortality rates in patients with HCC-NCL (Yen et al. 2021; Dasari et al. 2020; Penzkofer et al. 2022; Arnaoutakis et al. 2013; Verhoef et al. 2004; Schütte et al. 2014). While there may have been arguments against the impact of distant metastases and tumor infiltration on the survival of HCC-NCL patients (Grazi et al. 2003), deeper tumor infiltration and distant metastases are indicative of a higher extent of tumor progression. It is reasonable to assume that the more advanced the tumor, the worse the survival prognosis. Our analyses, including both univariate and multivariate models, showed that hepatectomy was a crucial independent risk factor affecting survival in accordance with previous research findings (Yen et al. 2021; Gawrieh et al. 2019; Wörns et al. 2012). Interestingly, we observed that local tumor destruction did not prolong survival time among patients with non-cirrhotic hepatocellular carcinoma. However, the roles of local ablative therapies like RFA and TACE in combination with selective internal radiotherapy (SIRT) for patients with HCC-NCL have yet to be fully determined (Desai et al. 2019). Additional studies are necessary to validate our results and establish the significance of our findings. AFP is a commonly used tumor marker for monitoring hepatocellular carcinoma (Hanif et al. 2022). However, there are varying opinions on its prognostic value in assessing patients with this disease, and higher levels of AFP have been shown to be a negative factor impacting the prognosis of HCC-NCL (Yen et al. 2021; Gawrieh et al. 2019; Yip et al. 2013; Penzkofer et al. 2022). Conversely, certain retrospective studies have suggested that AFP levels do not have an impact on long-term survival outcomes in cases of HCC-NCL (Demirtas et al. 2021; Verhoef et al. 2004; Schütte et al. 2014; Grazi et al. 2003). We propose that the contradictory outcome on AFP’s prognostic value may be attributed to discrepancies in AFP measurement methods and varying sample sizes (Hanif et al. 2022). In our study group, elevated AFP levels were associated with decreased patient survival, reinforcing the significance of AFP as a prognostic marker for overall survival in patients with HCC-NCL.

In the present study, Cox univariate analysis revealed that pathological grade was a prognostic risk factor for patients with HCC-NCL. However, when considered alongside other factors, the impact of pathological grade on survival duration did not reach statistical significance. These findings are consistent with previous retrospective studies (Penzkofer et al. 2022; Xu et al. 2008). Nonetheless, other studies have suggested that low differentiation tumors are associated with poorer prognosis in non-cirrhotic hepatocellular carcinoma patients (Gawrieh et al. 2019; Yip et al. 2013). The divergent results may be attributable to differences in classification criteria of non-cirrhotic hepatocellular carcinoma and sample sizes.

X-tile software has emerged as a common tool for identifying critical values, and we utilized it to develop a new risk stratification system based on nomogram. Our new risk stratification provides greater discriminatory ability in assessing patient risk and incorporates more influencing factors compared to the traditional AJCC staging system, leading to a more comprehensive evaluation of patient survival. Additionally, we conducted more rigorous statistical testing and model validation as well as included AFP, a widely employed clinical marker for hepatocellular carcinoma, in our study to confirm its prognostic value in patients with HCC-NCL, thus distinguishing our risk stratification system from those of previous studies (Dasari et al. 2020).

Yen et al. discovered that mortality increased by 10% for every 1-year rise in age (Yen et al. 2021). Another study confirmed that older age was associated with higher mortality rates among patients with HCC-NCL (Mohamad et al. 2015). However, studies by other investigators did not recognize age as an independent risk factor for death in cases of HCC-NCL (Dasari et al. 2020; Penzkofer et al. 2022; Grazi et al. 2003). In our research, age was not identified as a risk factor, nor were other factors such as gender, tumor number, preoperative low albumin, and ECOG physical status that were previously suggested to be correlated with overall survival in patients with HCC-NCL (Dasari et al. 2020; Penzkofer et al. 2022; Demirtas et al. 2021). Therefore, conducting meticulously designed prospective studies is imperative to thoroughly investigate and verify the factors that influence overall survival in patients with HCC-NCL.

Since this study was retrospective, it is possible that bias was introduced. First, the number of included liver transplant patients was small, leading to the inclusion of liver resection and liver transplantation in the same treatment group, potentially resulting in bias. Second, the grouping of NO and unknown chemotherapy information in the SEER database may generate some deviation as well. Finally, due to the utilization of the SEER database as the data source for this study, further external validation is necessary to confirm the conclusions drawn from this research. Additional research must be conducted to ascertain the generalizability and reliability of the study’s findings.

## Conclusion

In conclusion, utilizing significant risk factors identified from our analysis, we have established a practical and dependable nomogram for accurately predicting overall survival in non-cirrhotic hepatocellular carcinoma patients. Our approach also comprises a novel risk stratification tool that can assist clinicians in formulating appropriate clinical decisions for individual patients.

## Supplementary Information

Below is the link to the electronic supplementary material.

Supplementary file1 (XLSX 8 kb)

Supplementary file2 (XLSX 9 kb)

## Data Availability

The dataset analysis for this study can be found in the Surveillance, Epidemiology, and End Results (SEER) database.
